# Identification, systematic evolution and expression analyses of the AAAP gene family in *Capsicum annuum*

**DOI:** 10.1186/s12864-021-07765-1

**Published:** 2021-06-22

**Authors:** Xiaoxue Pan, Mingyu Hu, Zhongwei Wang, Ling Guan, Xiaoying Jiang, Wenqin Bai, Hong Wu, Kairong Lei

**Affiliations:** Biotechnology Research Center, Chongqing Academy of Agricultural Sciences/Chongqing Key Laboratory of Adversity Agriculture Research, Chongqing, 401329 China

**Keywords:** *Capsicum annuum*, Amino acid/auxin permease, Systematic evolution, Gene expression analyses

## Abstract

**Background:**

The amino acid/auxin permease (AAAP) family represents a class of proteins that transport amino acids across cell membranes. Members of this family are widely distributed in different organisms and participate in processes such as growth and development and the stress response in plants. However, a systematic comprehensive analysis of *AAAP* genes of the pepper (*Capsicum annuum*) genome has not been reported.

**Results:**

In this study, we performed systematic bioinformatics analyses to identify AAAP family genes in the *C. annuum* ‘Zunla-1’ genome to determine gene number, distribution, structure, duplications and expression patterns in different tissues and stress. A total of 53 *CaAAAP* genes were identified in the ‘Zunla-1’ pepper genome and could be divided into eight subgroups. Significant differences in gene structure and protein conserved domains were observed among the subgroups. In addition to *CaGAT1*, *CaATL4*, and *CaVAAT1*, the remaining *CaAAAP* genes were unevenly distributed on 11 of 12 chromosomes. In total, 33.96% (18/53) of the *CaAAAP* genes were a result of duplication events, including three pairs of genes due to segmental duplication and 12 tandem duplication events. Analyses of evolutionary patterns showed that segmental duplication of AAAPs in pepper occurred before tandem duplication. The expression profiling of the *CaAAAP* by transcriptomic data analysis showed distinct expression patterns in various tissues and response to different stress treatment, which further suggest that the function of *CaAAAP* genes has been differentiated.

**Conclusions:**

This study of *CaAAAP* genes provides a theoretical basis for exploring the roles of *AAAP* family members in *C. annuum*.

**Supplementary Information:**

The online version contains supplementary material available at 10.1186/s12864-021-07765-1.

## Background

Plants obtain nitrogen by absorbing ammonia, nitrate, amino acids, and soluble peptides from the soil. Nitrogen absorption and transport is mediated by several types of transport proteins, including ammonium transport proteins (AMTs), nitrate transport proteins (NRTs), amino acid transport proteins (AATs) and peptide transport proteins (PTRs) [1]. In plants, AATs are transmembrane (TM) proteins that transport amino acids from the extracellular environment to the intracellular environment [2]. According to conserved sequence and structure motifs, the plant AAT superfamily consists of the amino acid/auxin permease (AAAP) and amino acid-polyamine-choline (APC) gene families [3]. The AAAP subfamily includes eight subclasses of transporters: amino acid permeases (AAPs), lysine-histidine transporters (LHTs), proline transporters (ProTs), γ-aminobutyric acid transporters (GATs), putative auxin transporters (AUXs), similar to ANT1-like aromatic and neutral amino acid transporters (ANTs), and amino acid transporter-like (ATLa and ATLb) subfamilies [4, 5]. *AAAP* genes are widely present in plants, including *Arabidopsis* (46 genes) [6], rice (58 genes) [7], maize (71 genes) [8], poplar (71 genes) [9], potato (52 genes) [10], moso bamboo(55 genes) [11] and *Medicago truncatula*(86 genes) [12].

The first amino acid transporter protein (AtAAP1/NAT2) isolated from plants belongs to the AAP family. There are eight members in *Arabidopsis*, and AtAAP transports neutral, acidic and cationic amino acids with different specificities and affinities [13, 14]. *AtAAP1* is highly expressed in *Arabidopsis* cotyledons and the endosperm, and mediates uptake of amino acids to developing embryo or root cells [15–17]. *AtAAP2* is localized to the plasma membrane and the phloem, and the *aap2* mutant exhibits altered xylem-phloem transfer of amino acids, which affects metabolism and results in increased seed yield and oil content in *Arabidopsis* [18]. *AtAAP3* is exclusively expressed in roots and *AtAAP4* is primarily expressed in source leaves, stems, and flowers, *AtAAP5* has been observed in all tissues [19]. In the *aap6* mutant, the amino acid content of the *Arabidopsis* sieve elements was reduced but not affect leaves aphid herbivores [20]. *AtAAP8* participates in the early seed development in *Arabidopsis* [21]. *OsAAP3* and *OsAAP5* regulate tiller number and grain yield in rice [22, 23]**,** and overexpression of *OsAAP6* increases grain protein content and improves rice nutritional quality [24]. In addition, there are reports of AAP subfamily members in other species, including *StAAP1* [25], *PvAAP1* [26]*, PtAAP11* [27], *VfAAP1* and *VfAAP3* [28].

*AtLHT1* localizes on the surface of roots in young seedlings and in pollen and mediates uptake of amino acids from the root to the mesophyll cells through the xylem [29, 30]. Under conditions of nitrogen deficiency in particular, overexpression of *AtLHT1* can increase the efficiency of nitrogen utilization [30]. *AtLHT2* localizes to the tapetum of *Arabidopsis* anthers [31]. *AtLHT6* is expressed in buds, flowers, and roots; *AtLHT4* expression is increased in developed buds compared to mature flowers; and expression of *AtLHT5* peaks in flowers [32, 33]. *OsLHT6* is specifically expressed in new shoot meristems [7], and *PgLHT* plays an important role in the growth and development of the ginseng root system [34]. The GAT subfamily mainly transports γ-aminobutyric acid (GABA) and GABA-related compounds; the highest expression of *AtGAT1* is observed in flowers and under conditions of elevated GABA [35]. *AtANT1* is expressed in all organs, with the highest abundance in flowers and cauline leaves, and mediates transport of aromatic and neutral amino acids, arginine, indole-3-acetic acid, and 2, 4-dichlorophenoxyacetic acid [36]. *AtAUX1* is a high-affinity transporter of indoleacetic acid (IAA), and *AtAUX1* and *AtLAX3*(a homolog of *AtAUX1*) are mainly expressed in roots and promote lateral root formation [37, 38]. The expression of *OsAUX* subfamily members is also tissue-specific: *OsAUX4* is preferentially expressed in new shoot meristems, and *OsAUX2* and *OsAUX5* are specifically expressed in young roots, which suggests a role in the formation and development of root systems [7]. *MtLAX2*, a functional homolog of *AtAUX1*, is required for nodule organogenesis [39]. The ProTs subfamily is responsible for transporting proline, glycinebetaine (GB) and GABA. *AtProT1* is expressed in the phloem or phloem parenchyma cells, which indicates a role in the long-distance transport of proline [40]. By contrast, *AtProT2* is only expressed in root epidermis and cortical cells; *AtProT3* is more highly in leaf epidermal cells [40]. *HvProT2* is constitutively expressed in both leaves and roots, and heterologous expression experiments have shown that the affinity of *HvProT2* is highest for glycinebetaine [41]. *AtAVT3* and *AtAVT4* encode amino acid efflux proteins located in the vacuolar membrane, where they mediate transport of alanine and proline [42].

Pepper is an annual or perennial plant that belongs to the Solanaceae family; it is an important vegetable crop in China, which is number one in the world in terms of planting area and output (http://www.fao.org/faostat/en/). The pepper Zunla-1 (*C. annuum* L.) genome contains 34,476 protein-coding loci on 12 different chromosomes. Although the roles of many AAAPs in plants have been well characterized, members of the AAAP gene family in pepper have not been studied. We used bioinformatics to identify the AAAP gene family members in pepper and systematically analyzed the chromosome distribution, gene structure, evolution characteristics, and expression patterns of *AAAP* genes to provide a theoretical basis for exploring the roles of AAAPs in pepper.

## Results

### Identification of AAAP genes in pepper

To explore the AAAP protein family in pepper, we used one domain (PF01490) searche of Pepper Genome Database2 (http://peppersequence.genomics.cn/page/); the HMM profile was used as a query and each putative AAAP protein sequences was verified by SMART, CDD and Pfam analyses. A total of 53 AAAP genes were identified and renamed in pepper according to their affinities within gene subfamilies; *CaGAT1, CaANL4* and *CaVAAT1,* were not anchored to chromosomes (Table 1). Gene lengths ranged from 669 (*CaLHT4*) to 2532 bp (*CaAAP4*), the molecular weight varies from 24.43 kDa (*CaLHT4*) to 93.22 kDa (*CaAAP4*). The isoelectric points (pIs) of CaAAAP proteins ranged from 4.27(CaVAAT5) to 10.06(CaANT5); the majority of proteins (83%) had pIs more than 7.0, which indicates that AAAP proteins in pepper may represent a class of basic protein.

**Table 1:** The general information and sequence characterization of 53 *CaAAAP* genes

S.N.	Gene^**a**^	Locus^**b**^	Location^**c**^	ORF(bp)^**d**^	Exon^**e**^	Protein^**f**^			TM region^**g**^
						Size (aa)	MW(d)	pI	
	**AAP group**								
1	*CaAAP1*	Capana07g002429	Chr07:220179435–220,181,692	1335	7	444	49,460.8	8.72	11
2	*CaAAP2*	Capana07g002430	Chr07:220188828–220,192,330	1869	10	622	68,584.1	8.46	14
3	*CaAAP3*	Capana07g002431	Chr07:220195003–220,198,004	1410	7	469	51,763.2	8.45	10
4	*CaAAP4*	Capana07g002432	Chr07:220225817:220233681	2532	13	843	93,224.4	8.88	17
5	*CaAAP5*	Capana04g000780	Chr04:14469803:14475148	1446	7	481	52,757.7	8.81	10
6	*CaAAP6*	Capana12g000826	Chr12:27187513:27194231	1467	7	488	53,825.3	8.94	9
7	*CaAAP7*	Capana08g002210	Chr08:143014796:143019992	1419	7	472	51,747.8	9.12	11
8	*CaAAP8*	Capana04g001588	Chr04:67204663:67207202	1434	6	477	52,413.8	8.27	10
9	*CaAAP9*	Capana06g001752	Chr06:50038242:50040303	1419	6	472	51,681.9	7.84	10
10	*CaAAP10*	Capana05g001770	Chr05:174328892:174330262	1020	3	339	37,207.9	6.86	7
	**LHT group**								
11	*CaLHT1*	Capana02g003614	Chr02:162887482:162890584	1350	8	449	50,428	8.6	9
12	*CaLHT2*	Capana02g003615	Chr02:162905774:162912940	1266	7	421	47,443	8.24	9
13	*CaLHT3*	Capana02g003616	Chr02:162914284:162921151	1332	8	443	49,858.6	9.08	11
14	*CaLHT4*	Capana04g002888	Chr04:215599914:215604069	1227	9	408	46,113.8	8.27	7
15	*CaLHT5*	Capana04g001881	Chr04:130533897:130536648	1329	6	442	49,917.6	8.06	10
16	*CaLHT6*	Capana11g000230	Chr11:5761051:5762379	1329	1	442	49,944.5	9.1	10
17	*CaLHT7*	Capana03g001379	Chr03:25005836:25008812	1329	7	442	49,012.6	9.4	11
18	*CaLHT8*	Capana05g000336	Chr05:7406911:7414486	1065	7	354	39,858.5	9.42	7
19	*CaLHT9*	Capana11g002248	Chr11:216341951:216346445	1311	8	436	48,573.9	9.03	8
20	*CaLHT10*	Capana04g000478	Chr04:7738487:7744000	1581	5	526	57,977.4	9.61	9
21	*CaLHT11*	Capana04g000098	Chr04:1109665:1112218	1338	5	445	49,110.6	8.68	10
22	*CaLHT12*	Capana08g002793	Chr08:152269921:152272976	1713	5	570	61,963	9.55	9
23	*CaLHT13*	Capana11g000398	Chr11:11019799:11021033	708		235	25,865.9	9.01	2
24	*CaLHT14*	Capana04g000106	Chr04:1178475:1183677	669		222	24,427.3	8.47	3
	*GAT group*								
25	*CaGAT1*	Capana00g003418	Chr00:545297054:545303475	1365	7	454	49,950.5	8.68	10
26	*CaGAT2*	Capana11g000210	Chr11:5435275:5440152	1092	6	363	39,923.8	9.98	9
	**ProT group**								
27	*CaProT1*	Capana05g001989	Chr05:191409867:191415970	1320	7	439	47,836.8	9.73	12
28	*CaProT2*	Capana05g001990	Chr05:191424542:191430181	1347	7	448	49,162.1	9.4	11
29	*CaProT3*	Capana03g002827	Chr03:118029421:118036334	1344	7	447	49,190.2	9.61	12
	**AUX group**								
30	*CaAUX1*	Capana09g001555	Chr09:181029189:181033262	1467	7	488	54,841.3	8.15	10
31	*CaAUX2*	Capana10g001370	Chr10:147549183:147556929	1467	7	488	54,912.8	8.56	10
32	*CaAUX3*	Capana04g001744	Chr04:99262090:99266939	1317	8	438	49,663.3	8.25	9
33	*CaAUX4*	Capana08g002704	Chr08:150979738:150984984	1482	8	493	55,541.5	8.75	10
	**ANT group**								
34	*CaANT1*	Capana02g002432	Chr02:144978448:144979728	1281	1	426	46,665.9	7.92	11
35	*CaANT2*	Capana02g002433	Chr02:144981268:144982602	1335	1	444	48,548.7	4.74	11
36	*CaANT3*	Capana02g002434	Chr02:144983909:144985192	1284	1	427	46,457.5	4.82	11
37	*CaANT4*	Capana04g002414	Chr04:201839016:201840293	1278	1	425	46,811.9	7.45	11
38	*CaANT5*	Capana03g004210	Chr03:248829547:248830964	930	2	309	33,786.3	10.06	10
	**ATLa group**								
39	*CaATL1*	Capana06g001998	Chr06:75940086:75942122	846	3	281	30,550.9	4.94	3
40	*CaATL2*	Capana03g000522	Chr03:7178172:7179590	1419	1	472	51,153.5	5.43	10
41	*CaATL3*	Capana05g002081	Chr05:197860240:197862960	1302	6	433	47,459.1	8.35	11
42	*CaATL4*	Capana00g004937	Chr00:676629079:676631743	1320	5	439	47,898.6	8.55	11
43	*CaATL5*	Capana04g000715	Chr04:12477359:12484737	1344	5	447	48,657.5	8.36	11
44	*CaATL6*	Capana02g000804	Chr02:93929776:93933801	1407	5	468	50,795.2	8.78	11
45	*CaATL7*	Capana02g003206	Chr02:157224580:157228911	1383	5	460	49,954.5	8.55	11
	**ATLb group**								
46	*CaVAAT1*	Capana00g004212	Chr00:618994856:618996151	1296	1	431	46,618.7	7.96	8
47	*CaVAAT2*	Capana04g001726	Chr04:93008498:93010474	1281	3	426	46,958.9	7.71	10
48	*CaVAAT3*	Capana12g002556	Chr12:222093246:222094767	1017	2	338	36,992	7.91	9
49	*CaVAAT4*	Capana05g002349	Chr05:207916251:207920239	1140	3	379	41,865.5	9.04	9
50	*CaVAAT5*	Capana03g003057	Chr03:162840327:162847744	1395	9	464	51,484.7	4.27	7
51	*CaVAAT6*	Capana10g001696	Chr10:173666186:173669984	1608	11	535	57,908.7	5.19	10
52	*CaVAAT7*	Capana03g002859	Chr03:127734852:127743213	1338	7	445	48,690.8	4.98	5
53	*CaVAAT8*	Capana12g002523	Chr12:220748120:220761121	1989	15	662	73,214.4	5.85	8

S*.N* serial number, *ORF* open reading frame, *bp* base pair, *aa* amino acids, *MW* molecular weight, *pI* isoelectric point, *TM* transmembrane, *NA* not available

^a^Systematic designation given to pepper *AAAPs* in this study

^b^Locus identity number of AAAP assigned by Pepper Genome Database2 (http://peppersequence.genomics.cn/page/)

^c^Chromosomal localization of pepper AAAP genes

^d^Length of the open reading frame

^e^Number of extrons obtained from GSDS by comparing sequences between transcript and genome (Gene Structure Display Server; http://gsds.cbi.pku.edu.cn/)

^f^Protein characterization of CaAAAPs obtained from EXPASY server (http://web.expasy.org/protparam/)

^g^Number of transmembrane segments possessed by CaAAAPs, predicted by the TMHMM Server v2.0

**Fig. 1:**
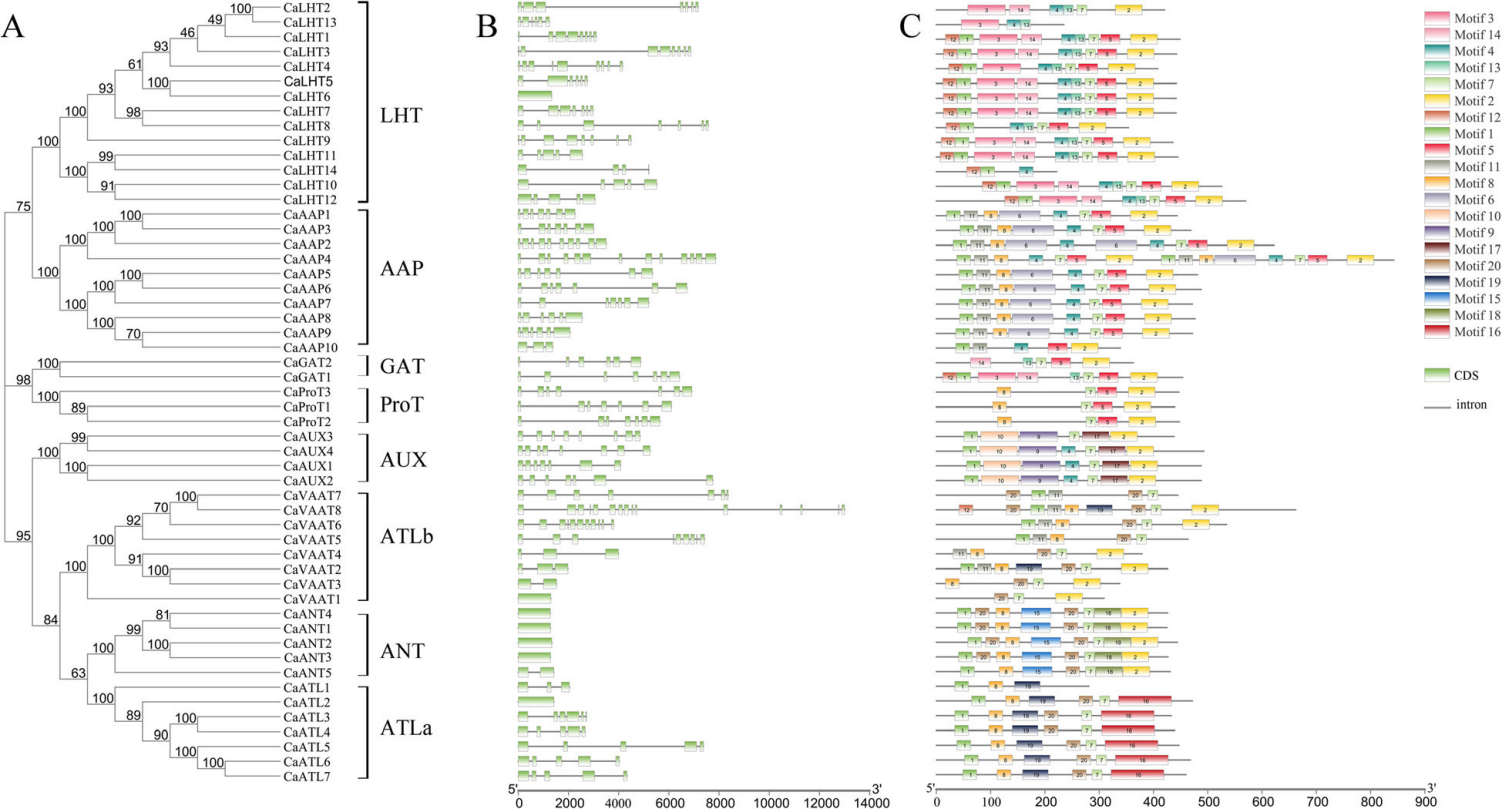
Phylogenetic relationship, gene structures and conserved motifs of CaAAAPs. **A** Phylogenetic tree of 53 CaAAAPs proteins. Neighbor-joining tree was constructed using MEGA7. Bootstrap support values from 1000 reiterations are indicated at each node. The 53 CaAAAPs in the tree were divided into eight subfamilies. **B** Exons and introns were indicated by green rectangles and gray lines respectively. **C** Conserved motifs of CaAAAPs proteins. Each colored box represents a specific motif in the protein identified using the MEME motif search tool. The order of the motifs corresponds to their position within individual protein sequences

We studied the exon/intron arrangement of the coding sequences of *CaAAAP* genes in their genome sequences and found that 13.21% (7/53) of pepper *AAAP* genes contained a single exon, 3.77% (2/53) had a single intron, and 83.02% had 1 to 14 introns (Fig. 1). Prediction of TM regions showed that most CaAAAPs (77.36%) had 8–11. Similar numbers of TMs regions were found in several subfamilies (e.g., 10 TMs in the AUX subfamily and 11 TMs in the ANT and ATLa subfamilies; Table 1 and Additional file 1:Figure S1). Thus, members of the same subfamily have a conserved structure.

Conserved domains of pepper AAAP proteins were analyzed with the MEME server and a total of 20 conserved motifs were identified (Fig. 1, Additional file 3: Table S1). Motifs 1 (44/53), 2 (42/53), and 7 (49/53) were widespread among members of the CaAAAP family. Some subfamilies included several specific motifs. For example, the LHT and GAT subfamilies contained motifs 3, 12, 13, and 14, whereas motif 5 was only found in the LHT, AAP, GAT, and ProT subfamilies. Motifs 9, 10, and 17 were only present in the AUX subfamily; motifs 15 and 18 were only present in the ANT subfamily; motifs 16 and 19 were only present in the ATLa subfamily. Similar numbers of motifs were found in the ProT and AUX subfamilies (Fig. 1), which suggests that the structures of these subfamilies are highly conserved.

### Phylogenetic and structural analyses of AAAP proteins in pepper

**Fig. 2:**
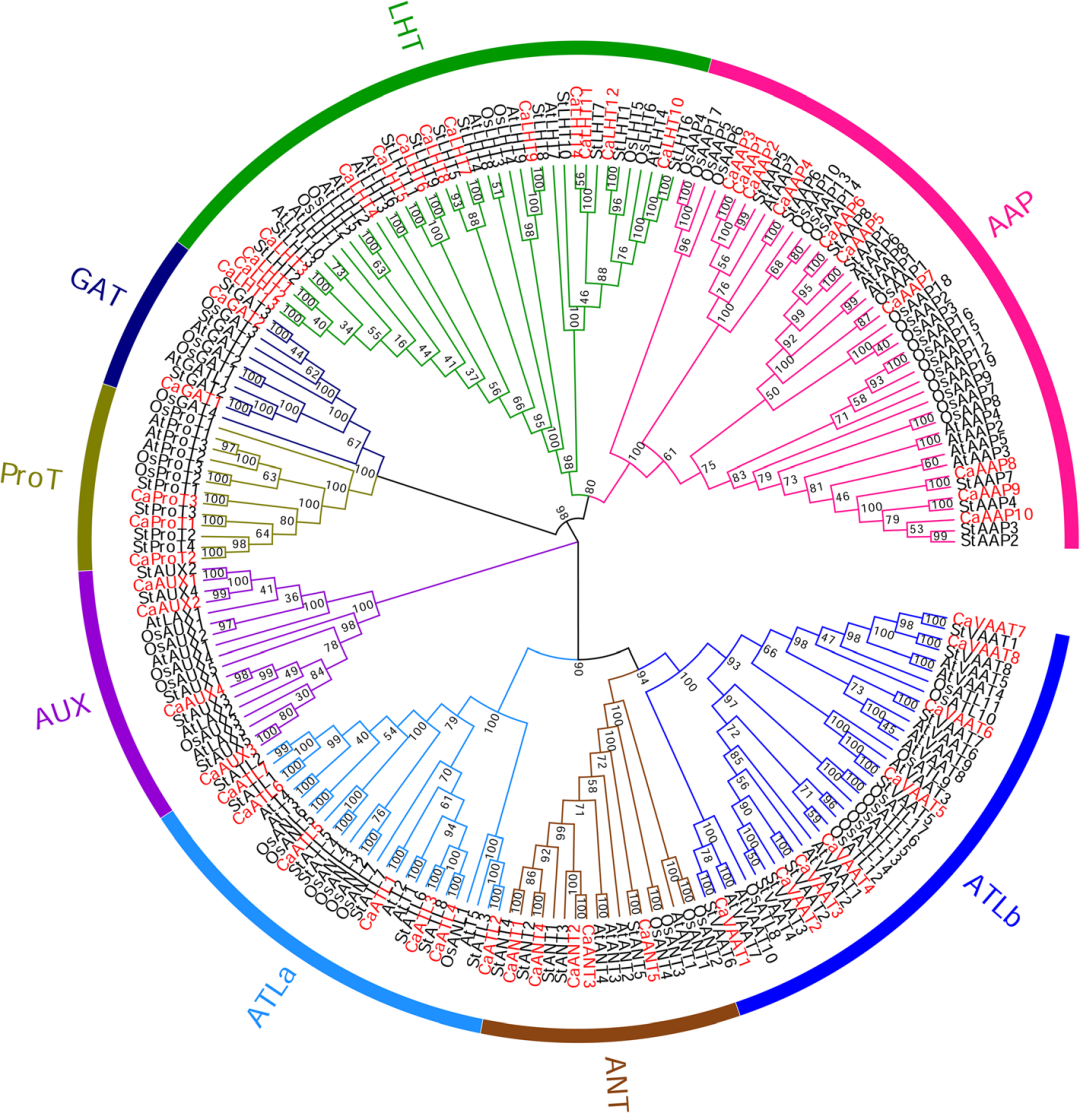
Phylogenetic relationships of pepper, potato, rice, and Arabidopsis AAAP proteins. Multiple sequence alignment of full-length proteins was performed by Clustal X1.83 and the phylogenetic tree was constructed using MEGA7 with the neighbor-joining method. The tree was divided into eight subgroups, marked by different color backgrounds

To further understand the homology between the AAAP gene families of pepper and other plant species (Table 2), we constructed an unrooted phylogenetic tree of full-length AAAPs from pepper, potato, rice and *Arabidopsis* was constructed (Fig. 2). We found that the genes *CaAAAP*, *StAAAP*, *OsAAAP* and *AtAAAP* were divided into eight distinct subfamilies, which indicates that the AAAP gene family has eight subfamilies in angiosperms. In pepper, the LHT subfamily was the largest (26.42%; 14 genes), whereas the GAT subfamily comprised only two genes. and the numbers of genes in the subgroups GAT, ProT,AUX and ANT were the same as or similar to those in potato, rice, and Arabidopsis.

**Table 2:** Comparative analysis of Amino acid/auxin permease (AAAP) proteins between Capsicum and other plant species

Specie	AAAP subfamily								# of AAAPproteins	# of Proteins	# % of AAAPproteins	Reference
	AAP	LHT	GAT	ProT	AUX	ANT	ATLa	ATLb				
*A. thaliana*	8	10	2	3	4	4	5	10	46	25,498	0.18	[6]
*P.edulis*	16	8	6	3	7	2	6	7	55	31,987	0.17	[11]
*O. sativa*	19	6	4	3	5	4	7	10	58	35,825	0.16	[7]
*Z. mays*	15	24	2	2	5	3	6	14	71	39,591	0.18	[8]
*M.truncatula*	26	18	4	3	5	3	13	14	86	44,623	0.19	[12]
*P. trichocarpa*	17	13	7	3	8	4	8	11	71	45,000	0.16	[9]
*S.tuberosum*	8	11	3	4	5	5	8	8	52	39,031	0.13	[10]
*C.annuum*	10	14	2	3	4	5	7	8	53	34,476	0.15	

*AAP* amino acid permease, *LHT* lysine and histidine transporter, *GAT* g -aminobutyric acid transporter, *ProT* proline transporter, *AUX* auxin transporter, *ANT* aromatic and neutral amino acid transporter, *ATL* amino acid transporter-like

### Chromosomal location and duplication analyses

**Fig. 3:**
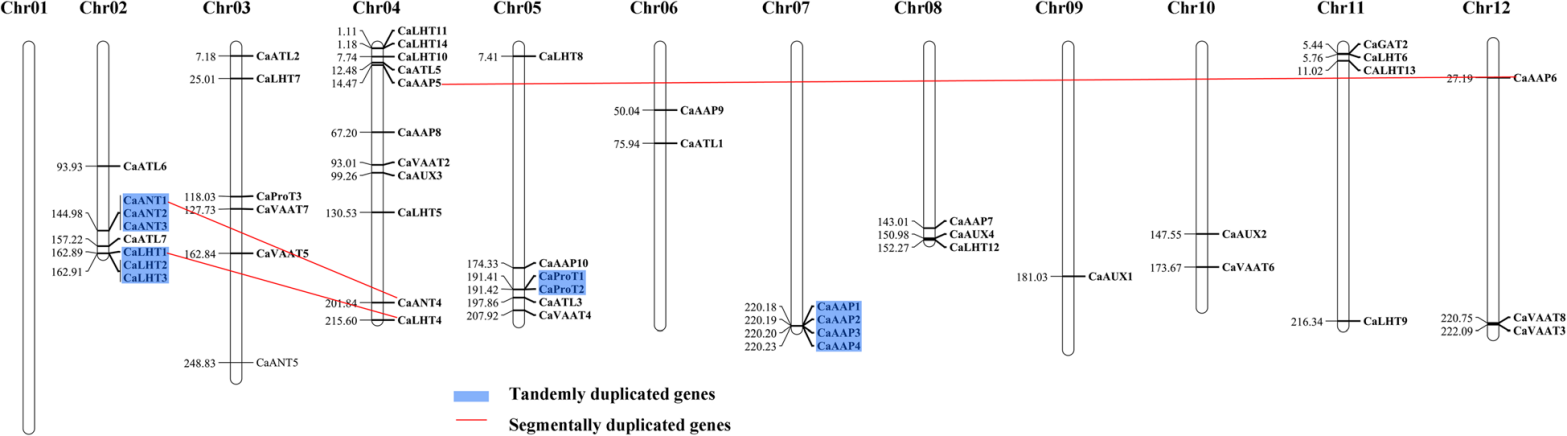
Chromosomal localization and gene duplication events of *CaAAAP* genes. Respective chromosome numbers are indicated at the top of each bar. Tandem duplicated genes are marked on a blue background. Segmental duplicated genes are shown by red line

We used Mapchart 2.30 mapping to identify the chromosomal location of AAAP genes in the pepper genome (Fig. 3). In addition to *CaGAT1, CaANL4* and *CaVATT1*, the remaining 50 genes were unevenly distributed on 11 of 12 chromosomes; no genes were mapped to chromosomes 1 (Fig. 3, Table 1). Most of the genes were mapped to the bottom of chromosomes 2, 5, 7 and 8, whereas the genes on chromosome 11 were mostly mapped to the top. A total of 58.5% (31/53) of genes were mapped to chromosome 2, 3, 4 and 5, which contained 8, 6, 11 and 6 genes, respectively. Only one gene was located on chromosome 9, and two to four genes were mapped to the remaining chromosomes (Fig. 3).

To identify the duplication events of *AAAP* genes in pepper, we analyzed the 53 full-length AAAP protein sequences using MCScanX. According to the defined criterion of separation five or fewer genes with more than 50% similarity at protein level, 33.96% (18 of 53) originated from the duplication events (Fig. 3). Twelve genes (22.64%) were arranged in tandem duplication and organized into four groups. Two pairs of tandem duplicate genes were identified on chromosome 2; chromosomes 5 and 7 each contained one pair (Fig. 3). Three segmental duplication blocks were located on chromosomes 2, 4 and 12, representing 11.32% of all *CaAAAP* genes (6/53) (Fig. 3, Additional file 2: Figure S2). Furthermore, high-sequence similarity occurred in duplicated genes: *CaAAP1* and *CaAAP3*, which originated via tandem duplication, were 94.28% similar, whereas *CaANT1* and *CaANT4*, which were a result of segmental duplication, exhibited 81.79% similarity.

Based on chromosomal distribution and phylogenetic and sequence similarity analyses, we identified seven pairs of paralogs in the pepper AAAP family (Table 3). Two pairs of paralogs (*CaANT1* and *CaANT4*, and *CaAAP5* and *CaAAP6*) participated in segmental duplications on different chromosomes. Five pairs (*CaANT1 and CaANT2*, *CaANT2 and CaANT3*, *CaANT1 and CaANT3*, *CaLHT1* and *CaLHT3*, and *CaAAP1 and CaAAP3*) were the result of a putative tandem duplication event. We further estimated nonsynonymous (Ka) and synonymous (Ks) nucleotide substitution rates in the coding sequences of paralog pairs to explore the selective pressures and duplication time of AAAP gene family members in pepper (Table 3). In general, Ka/Ks ratios less than 1 indicate purifying selection, and Ka/Ks ratios greater than 1 indicate positive selection [43]. The Ka/Ks ratios of all seven paralog pairs were < 1.0, which indicates that *CaAAAP* genes evolved under purifying selection (Table 3). We also estimated the dates of duplication events of paralog pairs using the formula T = Ks/2λ (assuming a clock-like rate (λ) of 6.96 × 10 ^− 9^ synonymous substitutions per years [44]); duplication events were estimated to have occurred 8.53 to 68.69 million years ago (Mya), with an average duplication time of 43.61 Mya. We estimate that the duplication time of two AAAP paralog pairs in pepper occurred 58.87 to 54 Mya and that of five of the paralogous gene pairs occurred 40.96 to 8.53 Mya (Table 3).

**Table 3:** Ka-Ks calculation for each pair of AAAP paralogs in pepper

Paralog pairs	S-sites	N-sites	Ka	Ks	Ka/Ks	Selection pressure	Duplication type	Duplication time (Mya)
*CaANT1-CaANT2*	304.25	970.75	0.16	0.57	0.29	Purifying selection	Tandem	40.96
*CaANT2-CaANT3*	305.00	976.00	0.05	0.12	0.40	Purifying selection	Tandem	8.53
*CaANT1-CaANT3*	304.83	970.17	0.15	0.57	0.26	Purifying selection	Tandem	40.59
*CaLHT1-CaLHT3*	313.75	1015.25	0.13	0.58	0.22	Purifying selection	Tandem	41.43
*CaAAP1-CaAAP3*	316.08	1015.92	0.07	0.14	0.50	Purifying selection	Tandem	10.37
*CaANT1-CaANT4*	303.58	971.42	0.11	0.82	0.14	Purifying selection	Segmental	58.87
*CaAAP5-CaAAP6*	351.08	1091.92	0.11	0.75	0.15	Purifying selection	Segmental	54.00

*S-Sites* number of synonymous sites, *N-Sites* number of non-synonymous sites, *Ka* non-synonymous substitution rate, *Ks* synonymous substitution, *Mya* million years ago

### Expression patterns of *CaAAAP* genes in various tissues

**Fig. 4:**
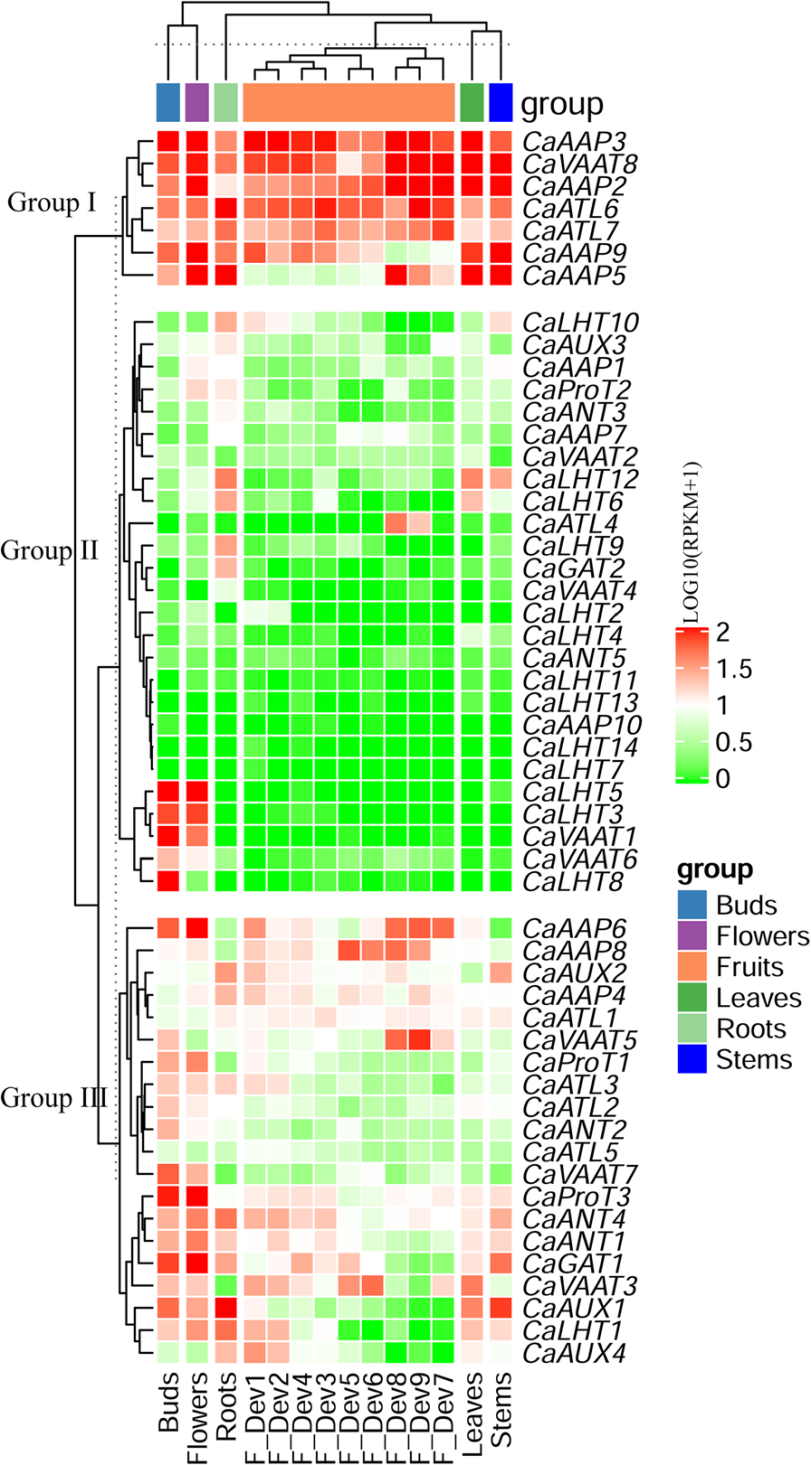
Expression profiles of *CaAAAP* genes in different tissues. The relative expression levels corresponding to log 10-transformed RPKM values after the addition of a pseudocount of 1 are shown. The scale represents the relative signal intensity of the RPKM values

We investigated the expression profiles of all *CaAAAP* genes in roots, stems, leaves, floral buds, flowers and different developmental stages of fruits (Fig. 4, Additional file 4: Table S2). 48 (90.5%) of the *CaAAAP* genes were detected in at least one tissue (RPKM ≥1), and 19(35.8%) genes were detected in all tissues tested (RPKM ≥1). In particular, approximately half of the *CaAAAP* genes showed low expression in fruits. By contrast, approximately 50% *CaAAAP* genes showed high expression in flowers and buds (RPKM ≥10). The *CaAAAP* genes clustered into three distinct clades based on expression patterns (Fig. 4). Seven genes (*CaAAP2, CaAAP3, CaAAP5*, *CaAAP9, CaATL6, CaATL7,* and *CaVAAT8*) in group I were expressed at relatively high levels in all tissues. In addition to several genes exhibited relatively high expression in specific organs (such as *CaLHT3*, *CaLHT5*, *CaLHT8, VAAT1* and *VAAT6* in buds; *CaATL4* in fruits; *CaLHT9* and *CaGAT2* in roots; *CaLHT12* in roots, stems and leaves), the other genes in group II were expressed at relatively low levels in all tested tissues. Group III comprised 20 genes that were expressed at relatively high levels in most organs.

### Differential expression profiling of *CaAAAP* genes in response to hormones and abiotic stress

**Fig. 5:**
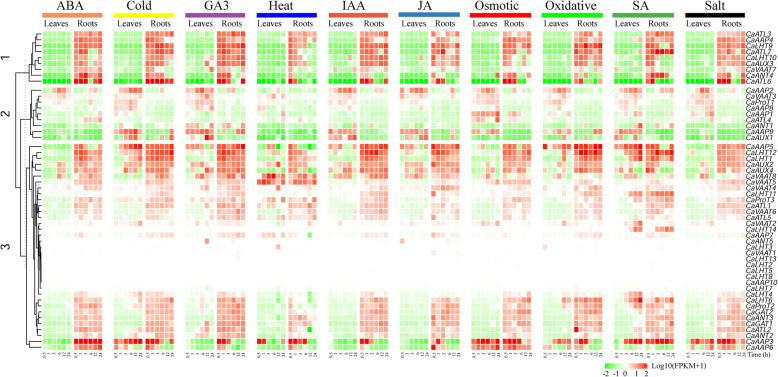
Expression patterns of *CaAAAP* genes in response to different hormone treatments and abiotic stress in the leaves and roots. Relative expression changes corresponding to log 10-transformed FPKM values between experimental and control tissues are shown. To avoid a situation where a FPKM equals 0, 1 was added to all the FPKM values. The scale represents the relative signal intensity of the FPKM values

To study whether *CaAAAPs* are involved in responses to hormones and abiotic stresses in pepper, we investigated the expression levels of the *CaAAPs* in the roots and leaves of 40-day old seedlings in response to cold, heat, salt, osmotic, oxidative, ABA, IAA, GA3, JA and SA treatment (Fig. 5, Additional file 5: Table S3). In addition to *CaLHT2*, *CaLHT5*, *CaLHT7*, *CaLHT8*, *CaLHT13*, and *CaAAP10*, most *AAAP* genes were induced in at least one of the treatment as compared with the control (Fig. 5). Interestingly, some *AAAP* genes varied greatly between the leaves and roots in the response to abiotic or hormones stress. For instance, *CaAAP4*, *CaLHT9*, *CaLHT10*, *CaATL3*, *CaATL6*, *CaATL7*, *CaAUX3*, and *CaVAAT7* were found to be upregulated under cold, heat, osomotic, oxidative and salt in the roots, but downregulated in the leaves. There were 28, 10, 20, and 18 *CaAAAP* genes were also upregulated by ABA, GA3, IAA, and JA treatment in the roots respectively, but downregulated in the leaves. Whereas there were 4, 5, and 7 *CaAAAP* genes were observed to be upregulated in the leaves but downregulated in the roots under the cold, IAA and salt stress treatment, respectively. In contrast, the highest number of *CaAAAP* genes were upregulated in the SA response in the leaves and roots (33 genes). There were several stress-responsive cis-elements showing in the promoter regions of these members, such as ABRE, ARE, LTR, MBS, TGACG-motif, CGTCA-motif, TCA-element, GARE-motif, AuxRR-core, and TC-rich repeats (Additional file 6: Table S4). Among the 53 AAAP genes, the *CaAAP7* promoter had no these stress-responsive elements, while *CaVAAT2* had maximum 14 elements. These results reaveled that a number of *CaAAAP* genes might involved in regulating abiotic and hormone stress responses.

## Discussion

The AAAP gene family, which contains eight subfamilies, encodes integral TM proteins that play a pivotal role in various aspects of normal plant growth and development. This gene family has been identified in many plants, including *Arabidopsis* [6], rice [7], maize [8], poplar [9], potato [10], moso bamboo [11] and *Medicago truncatula* [12]. Although the role of AAAP genes in plants has been previously suggested, systematic study of the AAAP gene family in pepper has not been performed. We identified 53 *CaAAAPs* genes in *C.annuum.Zunla-1* in this work. The number of *CaAAAPs* identified was similar to those in potato [10] and moso bamboo [11]. In addition, AAAP proteins account for 0.13 to 0.18% of the total proteins in many plant species studied (Table 2), and the percentage of CaAAAPs identified in the present study was 0.15%. Thus, the number of AAAP genes in most plants appears to be similar, regardless of genome size. Consistent with that in other plants, the pepper AAAP gene family can be divided into eight subfamilies (Fig. 2 and Table 2). Although the clade patterns are consistent with previous results from in other plants, the number of AAAP genes within sevearl subfamilies is significant difference (Table 2), which indicates that the expansion of each subfamily occurred after the split of dicot and monocot.

In addition to *CaGAT1, CaVATT1* and *CaATL4*, the remaining 50 genes were unevenly distributed on 11 of 12 chromosomes, and most of the genes were mapped on chromosomes 2, 3, 4 and 5 (Fig. 3). Meanwhile, four groups of tandem duplicate genes were identified on chromosome 2, 5 and 7, respectively, and segmental duplication blocks were located on chromosomes 2, 4 and 12 respectively (Fig. 3). In addition, gene structure analysis indicated the same subgroup had the same or similar numbers and types of exon/intron, TM regions, and motif compositions (Fig. 1, Table 1), which suggests that those groups have been relatively conserved during evolution.

Gene duplication is generally considered a major source of gene family expansion and functional diversity during evolution [45]. Previous studies also showed that 50% (29/58) of *AAAP* genes are duplicated gene in rice [7], duplicated genes represented 32.69% (17/52) in potato [10] and 30.43% (14/46) in Arabidopsis [6]. In the present study, 33.96% of *AAAP* genes (18/53) in pepper were duplicated genes, 12 genes (22.64%) are involved in the tandem duplication, and 6 genes (11.32%) in segmental duplication. These results suggest that tandem gene duplication is the main cause of expansion of the CaAAAP gene family; similar results have been reported in potato and Arabidopsis [6, 10]. The two pairs (*CaANT1* and *CaANT4*, and *CaAAP5* and *CaAAP6*) of paralogs participated in segmental duplications occurred from 54 to 58.87 Mya, and five pairs (*CaANT1 and CaANT2*, *CaANT2 and CaANT3*, *CaANT1 and CaANT3*, *CaLHT1* and *CaLHT3*, and *CaAAP1 and CaAAP3*) participated in tandem duplications occurred from 41.43 to 8.53 Mya (Table 3). This indicated that the segmental duplication of AAAPs in pepper occurred before tandem duplication. The pepper/potato separation occurred approximately 36 Mya [46], the duplication of most AAAP paralog pairs occurred before their separation from pepper and potato, and only two paralogous pairs were duplicated after the pepper/potato split. The Ka/Ks ratios of seven paralog pairs were < 1 (Table 3), which indicates that these paralog pairs evolved under purifying selection. Similar results have been reported in moso bamboo [11] and poplar [9], which have no paralog pairs in the AAAP family that underwent positive selection.

Gene duplication often causes changes in gene expression patterns and original functions of these genes may be retained [45]. Comparative analysis of the expression pattern of duplicated *CaAAAP* genes revealed that *CaANT2* and *CaANT3* (tandem duplicated genes) exhibited similar expression patterns in various development stages and stresses, which indicated that they may have overlapping functions (Figs. 4 and 5). However, most duplicated *CaAAAP* genes exhibited distinct expression patterns, such as *CaAAP5* and *CaAAP6* (segmental duplicated genes); as well as *CaAAP1* and *CaAAP3, CaLHT1* and *CaLHT3* (tandem duplicated genes) (Figs. 4 and 5). These results indicate that the expression and functional divergence of duplicated genes under selection pressure, contributing to adapt to the diversity of the environment.

Gene expression patterns are usually closely linked to plant growth and development, and comparative expression analyses of gene families can provide useful information for establishing their putative functions [47]. In this study, the expression profiles of *CaAAAP* differed across different organs and stages, consistent with the results of studies in other species such as potato [10]. Approximately 50% *CaAAAP* genes were expressed at relatively high levels in flowers and buds. 24 and 19 *CaAAAPs* genes showed relatively high expression levels in the roots and leaves, respectively. Similarly, 19 *StAATs* in potato were expressed at relatively high levels in the leaves [10]. Our data showed that *CaAAP5*, an orthologous of *StAAP1* and *AtAAP6*, was highly expressed in flowers, roots, leaves, and stems (Fig. 4). *AtAAP6* is responsible for the long-distance transport of amino acids [20]. *StAAP1*, which is highly expressed in leaves, stem, stolon and young tuber, is also responsible for the long-distance transport of amino acids [25]. Therefore, *CaAAP5* might be involved in the long-distance transport of amino acid in pepper. In *Arabidopsis*, *AtAUX1* and *AtLAX3* are highly expressed in roots [37, 48]. AUX subfamily genes are also mainly expressed in roots of rice and potato [7, 10]. In the study, AUX subfamily genes exhibited relatively high expression in roots, which indicates that *CaAUXs* might be involved in root growth and development. *CaATL4* was only expressed at a high level in F-Dev-8 and F-Dev-9, suggesting that *CaATL4* could play important roles in the late fruit developmen. Taken together, these results indicate that *CaAAAP*s may play an important role in the growth and development of pepper.

It has been reported that AAAPs is regulated by low temperature, high salt, and/or drought stress treatments in many plants [40, 49]. Under abiotic stress, 47 genes were regulated in at least one of the treatment as compared with the control and the expression of 48 genes were observed in all tissue analysis (Figs. 4 and 5). It has been reported that *HvProT* and *AtProT2* were strongly induced by salt stress [49, 50] . Similarly, we found that *CaProT1* had a close relationship with *AtProT2*, was specifically upregulated by cold, heat, salt, osmotic, oxidative, IAA, GA3, JA and SA stress in leaves. On the contrary, *AtAAP6* were found to be downregulated by salt stress [50]. CaAAP5, which is orthologous to *AtAAP6*, was downregulated under salt stress in leaves, but upregualtaed in roots. In moso bamboo, the AAP subfamily gene *PeAAAP9* has low expression level in leaf, but it is strongly induced by drought, cold and salt stress treatment [11]. Similary, *CaAAP6* was highly expressed under all ten stresses treatment in the roots. However, low expression of this gene was observed in root, suggesting that *CaAAP6* may take part in abiotic stress signaling pathways. With respect to the ten treatments, the expression of most *CaAAAP* was induced in the leaves or roots, suggesting that *CaAAAP* may play different roles in stress responses in pepper.

## Conclusions

Overall, 53 AAAP gene family members were identified in the ‘Zunla-1’ pepper genome and could be divided into eight subgroups. Throughout its evolutionary history, *CaAAAP*s were highly conserved and expanded slowly. *CaAAAP* genes exhibit tissue-specific expression and coordinate to regulate growth and development in pepper.

## Methods

### Data retrieval and identification of gene families

All pepper protein sequences were obtained from the Pepper Genome Database2 (http://peppersequence.genomics.cn/page/). The HMM profile for the AAAP domain (PF01490) downloaded from the Pfam database (http://pfam.xfam.org) [51], was used to identify potential AAAP genes from the pepper genome with HMMER 3.2.1 (http://hmmer.janelia.org/), with an E-value of 10^− 2^ [47]. BLAST analyses using the rice and Arabidopsis AAAPs as queries against the pepper genome with an E-value threshold of 10^− 10^. The sequences of the rice and Arabidopsis AAAP family were obtained from JGI (https://phytozome.jgi.doe.gov/pz/portal.html). After merging all of the putative pepper AAAP sequences, the candidate protein sequences were further verified for the presence of conserved domains with the online tools Conserved Domain Database (http://www.ncbi.nlm.nih.gov/cdd/), SMART (http://smart.embl-heidelberg.de/), and pfam (http://pfam.xfam.org/). The results were integrated and redundant genes were discarded. Molecular weights and pIs of the proteins encoded by the identified genes were predicted with the online EXPASY serve (http://web.expasy.org/protparam/).

### Phylogenetic tree, gene structure and conserved motif analyses of *CaAAAP* genes

Multiple sequence alignments analyses of AAAP amino acid sequences of *Arabidopsis*, rice, potato and pepper were performed with ClustalW. We built the phylogenetic tree using the neighbor-joining method with MEGA7 [52] and 1000 bootstrap replications, a Poisson model, and partial deletion gap parameters. We determined the exon/intron organization of *CaAAAP* genes by aligning the coding sequences with genomic sequences using the Gene Structure Display Server (http://gsds.cbi.pku.edu.cn/) [53]. Conserved motifs were generated with MEME (http://meme-suite.org/tools/meme) with the following parameters: zero or one motif in each sequence, 10 and 100 width of motifs, and a maximum of 20 motifs. Motifs were visualized with TBtools [54].

### Chromosomal location and syntenic analyses

The physical positions of the *CaAAAP* genes were obtained from the pepper annotation file deposited in the Sol Genomics database, mapped to 12 chromosomes, and visualized with Mapchart v.2.32 [55]. For syntenic analyses of *CaAAAP* genes, we used MCScanX [56] with the default settings to identify gene pairs of segmental and tandem duplications within the pepper genome.

### Expression patterns of *CaAAAP* genes in various tissues and different stresses

To study the expression patterns of pepper *AAAP* genes in the pepper plant, we downloaded transcriptome sequencing data from the NCBI (https://www.ncbi.nlm.nih.gov/geo/; accession no.GSE45037) [46]. These data covered a wide range of developmental stages of pepper: roots, stems and leaves from plants at the full-bloom stage; unopened flower buds (buds) and fully open flowers (flowers) from mature plants; and fruits lengths of 0–1, 1–3, 3–4, and 4–5 cm (F-Dev-1, F-Dev-1, F-Dev-3 and F-Dev-4, respectively); mature green fruit (F-Dev-5); fruit turning red (F-Dev-6); and fruit 3, 5, and 7 days after turning red (F-Dev-7, F-Dev-8, and F-Dev-9, respectively). A heat map representing digital expression profile of *CaAAAP* genes was created with R 3.6.3 with log-transformed values.

The gene expression data of pepper in roots and leaves under different stresses were downloaded from (http://pepperhub.hzau.edu.cn/) [57]. The 40-day-old seedlings were separately treated with 10 stress conditions in 0, 0.5, 1, 3, 6, 12 and 24 h: cold stress (10 °C), heat stress (42 °C), salt stress (200 mM NaCl), osmotic stress (400 mM D-mannitose), oxidative stress (30 mM H_2_O_2_), ABA stress (30 μM), IAA stress (2 μM), GA3 (2 μM), JA (10 μM) and SA (2 mM). A heat map representing digital expression profile of *CaAAAP* genes was created with R 3.6.3 with log-transformed values.

## Supplementary Information

**Additional file 1: Figure S1.** Prediction of the transmembrane regions of 53 CaAAAPs. The transmembrane regions of the 53 CaAAAPs were predicted using the TMHMM Server v2.0 (http://www.cbs.dtu.dkservicesTMHMM).

**Additional file 2: Figure S2.** Segmental duplication of 53 CaAAAPs. Gray lines indicate all synteny blocks in the pepper genome, the red lines indicate segmental duplicated genes.

**Additional file 3: Table S1.** MEME motif sequences and lengths of AAAP gene family proteins in pepper.

**Additional file 4: Table S2.** The RPKM expression values of *CaAAAP* genes at various developmental stages. These primary data was downloaded from NCBI, and then the relative expression level (log10 expression values) of 14 different tissues or evelopment stages was obtained after a series of manual processing.

**Additional file 5: Table S3.** Sample list and the FPKM data of *CaAAAP* genes under various stress treatment.

**Additional file 6: Table S4.** Summary of abiotic-stress inducible cis-elements in the promoter regions of AAAP genes in pepper.

## Data Availability

All the data obtained in the current study have been presented in this article.

## Abbreviations

RPKM: Reads Per Kilo bases per Million reads
FPKM: Fragments Per Kilo bases per Million reads

## References

[1] Williams LE, Miller AJ (2001). Transporters responsible for the uptake and partitioning of nitrogenous solutes. Annu Rev Plant Physiol Plant Mol Biol.

[2] Tegeder M (2012). Transporters for amino acids in plant cells: some functions and many unknowns. Curr Opin Plant Biol.

[3] Saier MH, Reddy VS, Tsu BV, Ahmed MS, Li C, Moreno-Hagelsieb G (2016). The transporter classification database (TCDB): recent advances. Nucleic Acids Res.

[4] Okumoto S, Pilot G (2011). Amino acid export in plants: a missing link in nitrogen cycling. Mol Plant.

[5] Fischer WN, André B, Rentsch D, Krolkiewicz S, Tegeder M, Breitkreuz K, Frommer WB (1998). Amino acid transport in plants. Trends Plantence.

[6] Rentsch D, Schmidt S, Tegeder M (2007). Transporters for uptake and allocation of organic nitrogen compounds in plants. FEBS Lett.

[7] Zhao H, Ma H, Yu L, Wang X, Zhao J (2012). Genome-wide survey and expression analysis of amino acid transporter gene family in rice (*Oryza sativa* L.). Plos One.

[8] Sheng L, Deng L, Yan HW, Zhao Y, Dong Q, Li Q, Li XY, Cheng BJ, Jiang HY. A genome-wide analysis of the AAAP gene family in maize. J Proteomics Bioinformatics. 2014; 07(1).

[9] Wu M, Wu SN, Chen Z, Dong Q, Yan HW, Xiang Y (2015). Genome-wide survey and expression analysis of the amino acid transporter gene family in poplar. Tree Genet Genomes.

[10] Ma HL, Cao XL, Shi SD, Li SL, Gao JP, Ma YL, Zhao Q, Chen Q (2016). Genome-wide survey and expression analysis of the amino acid transporter superfamily in potato (Solanum tuberosum L.). Plant Physiol Biochem.

[11] Liu HL, Wu M, Zhu DY, Pan F, Wang Y, Wang YJ, Xiang Y (2017). Genome-wide analysis of the AAAP gene family in moso bamboo (Phyllostachys edulis). BMC Plant Biol.

[12] Qu Y, Ling L, Wang D, Zhang T, Guo CH (2019). Genome-wide identification and expression analysis of the AAAP family in Medicago truncatula. Genetica..

[13] Fischer WN, Kwart M, Hummel S, Frommer WB (1995). Substrate specificity and expression profile of amino acid transporters (AAPs) in Arabidopsis. J Biol Chem.

[14] Okumoto S, Schmidt R, Tegeder M, Fischer WN, Rentsch D, Frommer WB, Koch W (2002). High affinity amino acid transporters specifically expressed in xylem parenchyma and developing seeds of Arabidopsis. J Biol Chem.

[15] Hirner B, Fischer WN, Rentsch D, Kwart M, Frommer WB (1998). Developmental control of H+/amino acid permease gene expression during seed development of Arabidopsis. Plant J.

[16] Lee YH, Foster J, Chen J, Voll LM, Weber AP, Tegeder M (2007). AAP1 transports uncharged amino acids into roots of Arabidopsis. Plant J.

[17] Sanders A, Collier R, Trethewy A, Gould G, Sieker R, Tegeder M (2009). AAP1 regulates import of amino acids into developing Arabidopsis embryos. Plant J.

[18] Zhang LZ, Tan QM, Lee R, Trethewy A, Lee YH, Tegeder M (2010). Altered xylem-phloem transfer of amino acids affects metabolism and leads to increased seed yield and oil content in Arabidopsis. Plant Cell.

[19] Okumoto S, Koch W, Tegeder M, Fischer WN, Biehl A, Leister D, Stierhof YD, Frommer WB (2004). Root phloem-specific expression of the plasma membrane amino acid proton co-transporter AAP3. J Exp Bot.

[20] Hunt E, Gattolin S, Newbury HJ, Bale JS, Tseng HM, Barrett DA, Pritchard J (2010). A mutation in amino acid permease AAP6 reduces the amino acid content of the Arabidopsis sieve elements but leaves aphid herbivores unaffected. J Exp Bot.

[21] Schmidt R, Stransky H, Koch W (2007). The amino acid permease AAP8 is important for early seed development in Arabidopsis thaliana. Planta..

[22] Lu K, Wu B, Wang J, Zhu W, Nie HP, Qian JJ, Huang WT, Fang ZM (2018). Blocking amino acid transporter OsAAP3 improves grain yield by promoting outgrowth buds and increasing tiller number in rice. Plant Biotechnol J.

[23] Wang J, Wu B, Lu K, Wei Q, Qian JJ, Chen YP, Fang ZM (2019). The amino acid permease 5 (OsAAP5) regulates tiller number and grain yield in rice. Plant Physiol.

[24] Peng B, Kong H, Li Y, Wang L, Zhong M, Sun L, Gao G, Zhang Q, Luo L, Wang G, Xie W, Chen J, Yao W, Peng Y, Lei L, Lian X, Xiao J, Xu C, Li X, He Y (2014). OsAAP6 functions as an important regulator of grain protein content and nutritional quality in rice. Nat Commun.

[25] Koch W, Kwart M, Laubner M, Heineke D, Stransky H, Frommer WB, Tegeder M (2003). Reduced amino acid content in transgenic potato tubers due to antisense inhibition of the leaf H+/amino acid symporter StAAP1. Plant J.

[26] Tan Q, Grennan AK, Hélène C, Pélissier RD, Tegeder M (2008). Characterization and expression of French bean amino acid transporter PvAAP1. Plant Sci.

[27] Couturier J, de Faÿ E, Fitz M, Wipf D, Blaudez D, Chalot M (2010). PtAAP11, a high affinity amino acid transporter specifically expressed in differentiating xylem cells of poplar. J Exp Bot.

[28] Miranda M, Borisjuk L, Tewes A, Heim U, Sauer N, Wobus U, Weber H (2001). Amino acid permeases in developing seeds of Vicia faba L.: expression precedes storage protein synthesis and is regulated by amino acid supply. Plant J.

[29] Chen L, Bush DR (1997). LHT1, a lysine- and histidine-specific amino acid transporter in arabidopsis. Plant Physiol.

[30] Hirner A, Ladwig F, Stransky H, Okumoto S, Keinath M, Harms A, Frommer WB, Koch W (2006). Affiliations expand Arabidopsis LHT1 is a high-affinity transporter for cellular amino acid uptake in both root epidermis and leaf mesophyll. Plant Cell.

[31] Lee YH, Tegeder M (2004). Selective expression of a novel high-affinity transport system for acidic and neutral amino acids in the tapetum cells of Arabidopsis flowers. Plant J.

[32] Perchlik M, Foster J, Tegeder M (2014). Different and overlapping functions of Arabidopsis LHT6 and AAP1 transporters in root amino acid uptake. J Exp Bot.

[33] Foster J, Lee YH, Tegeder M (2008). Distinct expression of members of the LHT amino acid transporter family in flowers indicates specific roles in plant reproduction. Sex Plant Reprod.

[34] Zhang R, Zhu J, Cao HZ, Xie XL, Hung JJ, Chen XH, Luo ZY (2013). Isolation and characterization of LHT-type plant amino acid transporter gene from Panax ginseng Meyer. J Ginseng Res.

[35] Meyer A, Eskandari S, Grallath S, Rentsch D (2006). AtGAT1, a high affinity transporter for gamma-aminobutyric acid in Arabidopsis thaliana. J Biol Chem.

[36] Chen L, Ortiz-Lopez A, Jung A, Bush DR (2001). ANT1, an aromatic and neutral amino acid transporter in Arabidopsis. Plant Physiol.

[37] Ugartechea-Chirino Y, Swarup R, Swarup K, Péret B, Whitworth M, Bennett M (2010). The AUX_1_ LAX family of auxin influx carriers is required for the establishment of embryonic root cell organization in Arabidopsis thaliana. Ann Bot.

[38] Marchant A, Kargul J, May ST, Muller P, Delbarre A, Perrot-Rechenmann C, Bennett MJ (1999). AUX1 regulates root gravitropism in Arabidopsis by facilitating auxin uptake within root apical tissues. EMBO J.

[39] Roy S, Robson F, Lilley J, Liu CW, Cheng X, Wen J, Walker S, Sun J, Cousins D, Bone C, Bennett MJ, Downie JA, Swarup R, Oldroyd G, Murray JD (2017). MtLAX2, a functional homologue of the Arabidopsis auxin influx transporter AUX1, is required for nodule organogenesis. Plant Physiol.

[40] Grallath S, Weimar T, Meyer A, Gumy C, Suter-Grotemeyer M, Neuhaus JM, Rentsch D (2005). The AtProT family. Compatible solute transporters with similar substrate specificity but differential expression patterns. Plant Physiol.

[41] Fujiwara T, Mitsuya S, Miyake H, Hattori T, Takabe T (2010). Characterization of a novel glycinebetaine/proline transporter gene expressed in the mestome sheath and lateral root cap cells in barley. Planta..

[42] Fujiki Y, Teshima H, Kashiwao S, Kawano-Kawada M, Ohsumi Y, Kakinuma Y, Sekito T (2017). Functional identification of AtAVT3, a family of vacuolar amino acid transporters, in Arabidopsis. FEBS Lett.

[43] Koonin EV, Rogozin IB (2003). Getting positive about selection. Genome Biol.

[44] Moniz de Sá M, Drouin G (1996). Phylogeny and substitution rates of angiosperm actin genes. Mol Biol Evol.

[45] Kong H, Landherr LL, Frohlich MW, Leebens-Mack J, Ma H, dePamphilis CW. Patterns of gene duplication in the plant SKP1 gene family in angiosperms: evidence for multiple mechanisms of rapid gene birth. Plant J 2007;50(5):873–885, doi: 10.1111/j.1365-313X.2007.03097.x. 10.1111/j.1365-313X.2007.03097.x 17470057

[46] Qin C, Yu C, Shen Y, Fang X, Chen L, Min J, Cheng J, Zhao S, Xu M, Luo Y, Yang Y, Wu Z, Mao L, Wu H, Ling-Hu C, Zhou H, Lin H, Gonzalez-Morales S, Trejo-Saavedra DL, Tian H, Tang X, Zhao M, Huang Z, Zhou A, Yao X, Cui J, Li W, Chen Z, Feng Y, Niu Y, Bi S, Yang X, Li W, Cai H, Luo X, Montes-Hernandez S, Leyva-Gonzalez MA, Xiong Z, He X, Bai L, Tan S, Tang X, Liu D, Liu J, Zhang S, Chen M, Zhang L, Zhang L, Zhang Y, Liao W, Zhang Y, Wang M, Lv X, Wen B, Liu H, Luan H, Zhang Y, Yang S, Wang X, Xu J, Li X, Li S, Wang J, Palloix A, Bosland PW, Li Y, Krogh A, Rivera-Bustamante RF, Herrera-Estrella L, Yin Y, Yu J, Hu K, Zhang Z (2014). Whole-genome sequencing of cultivated and wild peppers provides insights into Capsicum domestication and specialization. Proc Natl Acad Sci U S A.

[47] Liu AK, Liu CL, Lei HY, Wang ZJ, Zhang M, Yang XR, Yang G, Ren JH (2020). Phylogenetic analysis and transcriptional profiling of WRKY genes in sunflower (*Helianthus annuus* L.): genetic diversity and their responses to different biotic and abiotic stresses. Ind Crops Products.

[48] Yang Y, Hammes UZ, Taylor CG, Schachtman DP, Nielsen E. High-affinity auxin transport by the AUX1 influx carrier protein [published correction appears in Curr biol. Curr Biol 2006;16(11):1123–1127, doi: 10.1016/j.cub.2006.04.029. 10.1016/j.cub.2006.04.029 16677815

[49] Ueda A, Shi W, Sanmiya K, Shono M, Takabe T (2001). Functional analysis of salt-inducible proline transporter of barley roots. Plant Cell Physiol.

[50] Rentsch D, Hirner B, Schmelzer E, Frommer WB (1996). Salt stress-induced proline transporters and salt stress-repressed broad specificity amino acid permeases identified by suppression of a yeast amino acid permease-targeting mutant. Plant Cell.

[51] El-Gebali S, Mistry J, Bateman A (2019). The Pfam protein families database in 2019. Nucleic Acids Res.

[52] Kumar S, Stecher G, Tamura K (2016). MEGA7: molecular evolutionary genetics analysis version 7.0 for bigger datasets. Mol Biol Evol.

[53] Hu B, Jin J, Guo AY, Zhang H, Luo J, Gao G (2015). GSDS 2.0: an upgraded gene feature visualization server. Bioinformatics..

[54] Chen CJ, Chen H, Zhang Y, Thomas HR, Frank MH, He YH, Xi R (2020). TBtools: an integrative toolkit developed for interactive analyses of big biological data. Mol Plant.

[55] Voorrips RE (2002). MapChart: software for the graphical presentation of linkage maps and QTLs. J Hered.

[56] Wang Y, Tang H, Debarry JD (2012). MCScanX: a toolkit for detection and evolutionary analysis of gene synteny and collinearity. Nucleic Acids Res.

[57] Liu F, Yu H, Deng Y, Zheng J, Liu M, Ou L, Yang B, Dai X, Ma Y, Feng S, He S, Li X, Zhang Z, Chen W, Zhou S, Chen R, Liu M, Yang S, Wei R, Li H, Li F, Ouyang B, Zou X (2017). PepperHub, an informatics hub for the chili pepper research community. Mol Plant.

