# Supplementary figures and images for: Identification, systematic evolution and expression analyses of the AAAP gene family in Capsicum annuum
Source: BMC Genomics. 2021 Jun 22;22:463. doi: 10.1186/s12864-021-07765-1 (PMC8218413; doi:10.1186/s12864-021-07765-1)

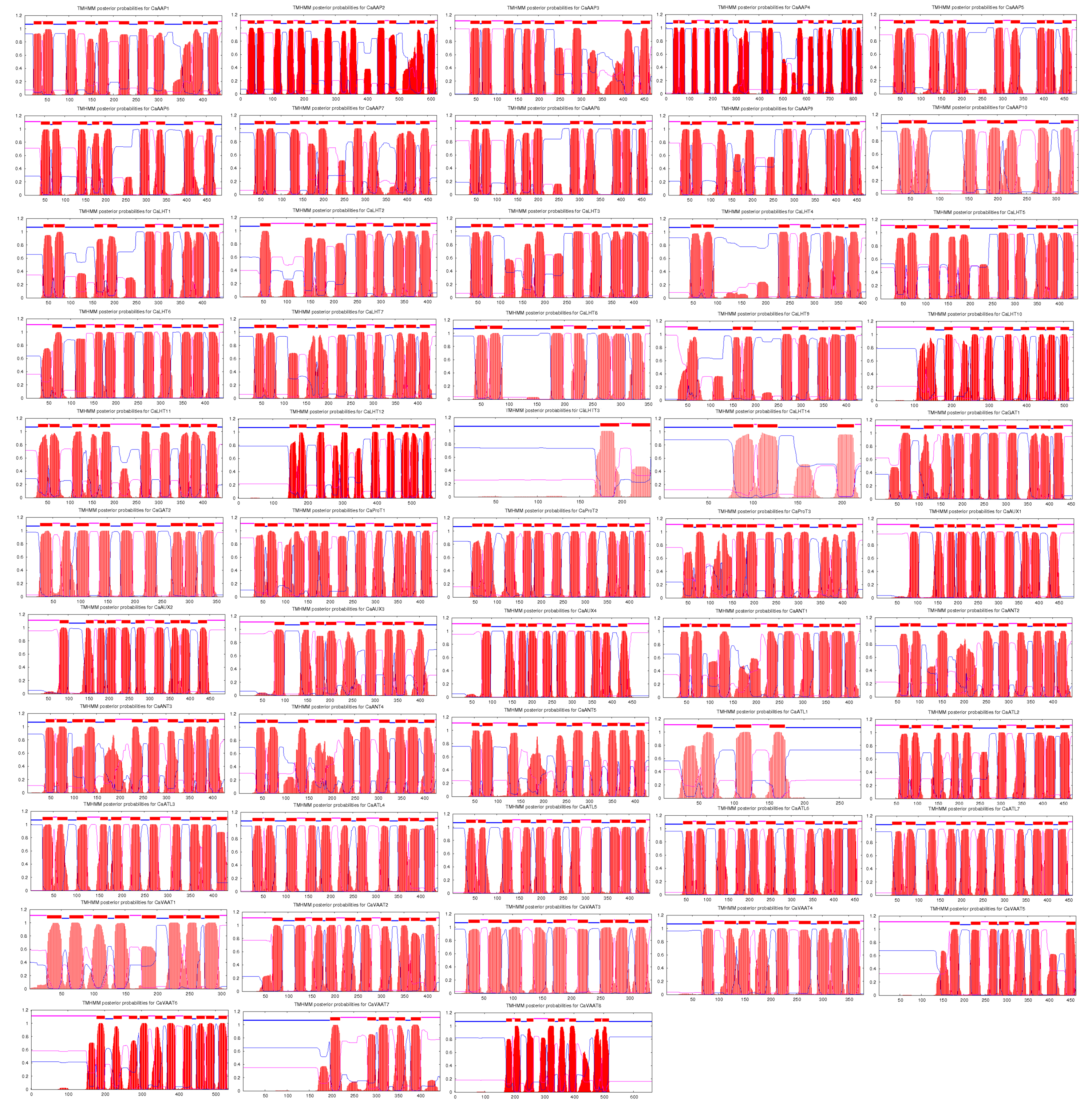

Supplement: Supplementary file 1 — Additional file 1: Figure S1. Prediction of the transmembrane regions of 53 CaAAAPs. The transmembrane regions of the 53 CaAAAPs were predicted using the TMHMM Server v2.0 (http://www.cbs.dtu.dkservicesTMHMM). [file 12864_2021_7765_MOESM1_ESM.tif]

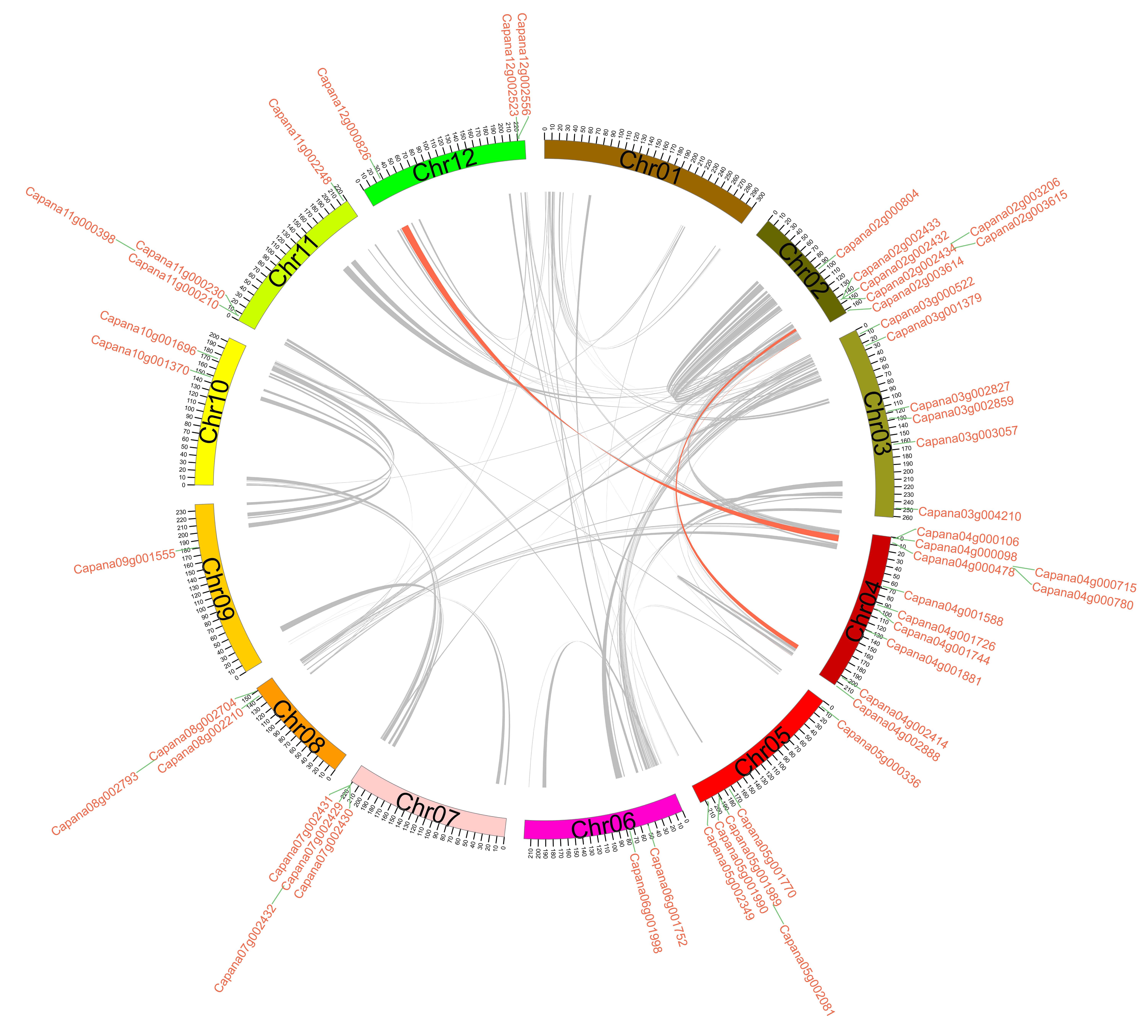

Supplement: Supplementary file 2 — Additional file 2: Figure S2. Segmental duplication of 53 CaAAAPs. Gray lines indicate all synteny blocks in the pepper genome, the red lines indicate segmental duplicated genes. [file 12864_2021_7765_MOESM2_ESM.tif]
