# Supplementary material for: Identification, systematic evolution and expression analyses of the AAAP gene family in Capsicum annuum
Source: BMC Genomics. 2021 Jun 22;22:463. doi: 10.1186/s12864-021-07765-1 (PMC8218413; doi:10.1186/s12864-021-07765-1)
Supplement: Supplementary file 3 — Additional file 3: Table S1. MEME motif sequences and lengths of AAAP gene family proteins in pepper. [file 12864_2021_7765_MOESM3_ESM.doc]

Table S1. The MEME motif sequences and lengths of AAAP gene family proteins in pepper.

| Motif | Width | Best possible match |
| --- | --- | --- |
| 1 | 26 | HIITAVVGAGVLSLPYAMAQLGWVAG |
| 2 | 36 | ALLFPFFGDLLGLVGAFLFAPLTYYLPCLMYJVIKK |
| 3 | 26 | LGDIAFAYAGHNVVLEIQATLKSPPK |
| 4 | 39 | LFGFYEPFWLIDFANACIVVHLIGSYQVYAQPVFAFVEK |
| 5 | 66 | TLYTLWQMVEMHEMVPGKRFDRYHELGQHAFGEKLGLWIVVPQQLVVEVGTDIVYMVTGGKSLKKF |
| 6 | 112 | KRNYTYMDAVRANLGGKKVWJCGLLQYVNLVGVAIGYTITSSISMVAIQRSNCFHKHGHKAHCHVSSTPYMIIFGVIZIILSQIPBFHNMSWLSIVAAVMSFTYSTIGLGLG |
| 7 | 34 | LGAATLFWNYNGFDAVSTLAEEVKNPKKDLPKGL |
| 8 | 127 | YGTPLIGILFSASGVJLLSWMSFQEIVAAENFLYSFGMJLEFIAFVWLRIKFPDAKRPYKIPGGTVGAVLLCJPPTILJVVVMVLASIKVMIVSLLAIAIGJVMYPCLKLIEKKKWLKFSTSSDLPD |
| 9 | 19 | YLPVAVFGYWAFGBSTPDN |
| 10 | 114 | EMKKSLTWWDLVWFGIGAVIGAGIFVLTGQEAREHAGPAVVLSYAISGISALLSVFCYTEFAVEIPVAGGSFAYLRVELGEFVAFIAAGNILLEYVIGGAAVARSWTSYFATLL |
| 11 | 102 | GMNWAKYVVAIGALKGMTTVLLVGAIGQARYLTHIARTHMIPPWFAQVHPKTGTPVNATLIMGJATAVJALFTSLDILSNLLSIGTLFVFSLVALALLVRRY |
| 12 | 53 | PLLAJLGFLIFPLIWSVPEALITAELGTTFPENGGYVVWVSSALGPYWGFQQG |
| 13 | 98 | WKWLSGVIDNALYPVLFLDYLKSAIPILGGGLPRIJAILGITLVLTYLNYRGLTIVGWVAVALGILSJLPFIVMGLISIPKJRPSRWLVLDVKNVEWB |
| 14 | 36 | DJGFAAFGKVGRILVQICIVLENYGVLVGYLIIIGD |
| 15 | 50 | GGVWLKWWIZGAAALSNMGLFEAEMSSDSFQLLGMAERGMLPEFFAKRSR |
| 16 | 70 | CASNIYYINDNLDKRTWTYIFGACCATTVFIPSFHNYRIWSFLGLGMTTYTAWYLTIAALJHGQVEGVKH |
| 17 | 195 | HEVSNDSWFQVGIVLSTGINSAYALGYAGTIMVPLGWJGGVVGLVLSTJISLYASTLMAKLHEYGGKRHIRYRDLAGFJYGRTAYJLVWALQYANLFLINIGFVILGGQALKAFYVLFRDDHQMKLPHFIAIAGFACVLFAIAVPHLSALRVWLGFSSLFSLIYLTIAFVLSLKDGIKAPPRDYSIPGSKDNKIW |
| 18 | 98 | SRYFVJLLTTLFVFAPLVSYKRIDSLRLSSALSVALAVVFVVITVGIAIIKLINGSIVMPRLLPDLYDVTSFLKLFTVVPVLVTAYICHYNVHPIGNE |
| 19 | 26 | JVLFLFAFVTYYTSMLLSDCYRSSDP |
| 20 | 41 | NCKPJKLTYFILIFTSVHFVLSQLPNFNSISGVSLAAAVMS |
